# A Follow-Up Content Analysis of Aging Network Conference Proceedings: Aligning Research and Practice

**DOI:** 10.1093/geroni/igaa057.207

**Published:** 2020-12-16

**Authors:** Todd Becker, Rajean Moone, Joan Davitt

**Affiliations:** 1 University of Maryland, Baltimore, Baltimore, Maryland, United States; 2 University of Minnesota, Woodbury, Minnesota, United States

## Abstract

The federal government established a collective of agencies tasked with providing support and services to older adults and their caregivers known as the Aging Network. In order to effectively provide these services, the Aging Network must identify and disseminate current best practices. To this end, the Aging Network hosts three annual conferences: the National Association of Area Agencies on Aging (n4a), the Alliance of Information and Referral Systems (AIRS), and the National Association of States United for Aging and Disabilities (NASUAD). This content analysis of key themes emerging from Aging Network conference abstracts (N = 2,392) from 2009 to 2019 expanded upon a similar study analyzing the preceding decade of conference materials (Moone & Cagle, 2011). Reflexivity, analyst triangulation, and confirmability and dependability audits were used to enhance trustworthiness. The most common themes included planning and program development (n = 260), general policy (n = 166), and long-term services and supports system reform (n = 162). Although some themes, such as consumer-directed support (n = 122) and advocacy (n = 111) were consistent with the original article (Moone & Cagle, 2011), others, such as managed care (n = 124) and financial exploitation (n = 50), demonstrated a societal shift in older adult supports and services. These results offer an additional effort towards disseminating current best practices and emerging issues to advance translation between research and practice. Researchers can use these results to better align their research agendas with the needs of the Aging Network for evidence-based interventions.

